# Case Report: a hypermetabolic mediastinal mass (SUVmax 16.7) mimicking malignancy with spontaneous resolution: an inflammatory myofibroblastic proliferation and diagnostic pitfall

**DOI:** 10.3389/fonc.2026.1901288

**Published:** 2026-08-05

**Authors:** Zhe Li, Zhuo Zhang, Baolei Lyu

**Affiliations:** 1 Department of Thoracic Surgery, Shijiazhuang People’s Hospital, Shijiazhuang, China; 2 Department of Pathology, Shijiazhuang People’s Hospital, Shijiazhuang, China

**Keywords:** 18F-FDG PET/CT, CT-guided core biopsy, diagnostic pitfall, inflammatory myofibroblastic proliferation, Ki-67, mediastinal mass, retrotracheal, spontaneous resolution

## Abstract

**Background:**

Hypermetabolic mediastinal masses on 2-deoxy-2-[18F]fluoro-D-glucose ([18F]FDG) positron emission tomography/computed tomography (PET/CT) are presumed malignant until proven otherwise. However, benign inflammatory and organizing processes may exhibit equally intense metabolic activity, posing a diagnostic challenge—particularly when the lesion abuts major vascular structures and limits safe tissue sampling.

**Case summary:**

A 55-year-old male smoker presented with cough, right-sided chest pain, low-grade fever, and mild dysphagia. Contrast-enhanced CT identified an ill-defined mass in the retrotracheal region of the superior mediastinum (within the tracheoesophageal groove), measuring 1.8×2.6×3.2 cm, with poorly defined margins against the esophagus and heterogeneous enhancement (precontrast attenuation 17–41 Hounsfield units (HU)). [18F]FDG PET/CT demonstrated markedly elevated [18F]FDG uptake (maximum standardized uptake value [SUVmax], 16.7), raising strong suspicion for malignancy. Serum tumor markers, procalcitonin, interleukin-6, and total immunoglobulin E (IgE) were within normal limits; sputum acid-fast, fungal, and bacterial cultures were negative, as were Epstein–Barr virus, cytomegalovirus, and a respiratory virus panel. Endobronchial ultrasound (EBUS)-guided aspiration did not yield a diagnostic specimen. CT-guided core needle biopsy was safely performed despite proximity to the superior vena cava and aortic arch, without complications. Histopathology revealed fibrocollagenous tissue with lymphoplasmacytic infiltration and foamy histiocytes, without malignant cells. Immunohistochemistry showed anaplastic lymphoma kinase (ALK) negativity, smooth muscle actin (SMA) positivity, and immunoglobulin G4 (IgG4) sparse focal weak positivity (below the threshold for IgG4-related disease), and a Ki-67 index of approximately 30% that labeled predominantly inflammatory cells rather than spindle cells, consistent with an inflammatory myofibroblastic proliferation. No antineoplastic, further targeted antimicrobial, or immunosuppressive therapy was administered. Follow-up CT at approximately 2.5 months demonstrated complete spontaneous resolution of the mass. A repeat chest CT in July 2026, approximately 2 months after the scan that first documented complete resolution, continued to show no residual or recurrent mediastinal lesion. Taken together, the findings favored an inflammatory myofibroblastic proliferation with an organizing, self-limiting course, while acknowledging that complete distinction from an ALK-negative inflammatory myofibroblastic tumor cannot be made with absolute certainty on limited core-biopsy material alone.

**Conclusion:**

An inflammatory myofibroblastic proliferation in the mediastinum may exhibit extreme FDG avidity (SUVmax 16.7) indistinguishable from malignancy on imaging. A Ki-67 index of ~30% that labels predominantly inflammatory cells supports reactive proliferative activity as a histological correlate of the elevated metabolic signal, whereas complete spontaneous resolution sustained on repeat CT supports a self-limiting inflammatory course. When tissue sampling and subsequent clinical evolution make malignancy and active infection less likely, short-interval imaging surveillance may be an appropriate strategy in carefully selected patients.

## Introduction

On [18F]FDG PET/CT, intense FDG uptake in a mediastinal mass is broadly interpreted as a marker of malignancy, with an SUVmax substantially above background carrying a high positive predictive value in published series (1, 2). This interpretation, however, is limited by the non-specificity of glucose metabolism: active inflammatory and organizing processes—rich in macrophages, activated lymphocytes, and proliferating myofibroblasts—can produce equally intense FDG accumulation and thereby mimic malignancy (3, 4).

This diagnostic difficulty is compounded when the lesion lies in close proximity to major vascular structures, where percutaneous biopsy carries significant risk and may yield limited material (5, 6). The clinician is then faced with a metabolically alarming lesion, an anatomically hazardous biopsy target, and a broad differential spanning malignancy, infection, and immune-mediated or idiopathic inflammation.

We report a mediastinal mass with an exceptionally high SUVmax of 16.7 that was initially considered malignant, in which a carefully planned percutaneous core biopsy revealed an inflammatory myofibroblastic proliferation, and which subsequently underwent complete spontaneous resolution without any targeted treatment. The case illustrates an important diagnostic pitfall and provides a histological correlate—an elevated reactive Ki-67 index—for the unexpectedly high metabolic activity.

## Case description

### Clinical presentation

A 55-year-old man with a 20 pack-year smoking history (ceased prior to presentation) presented with a one-month history of intermittent dry cough, right-sided pleuritic chest pain, low-grade fever (maximum 37.8 °C), and mild dysphagia. Empirical antibiotics at a community hospital had produced only partial symptomatic improvement. The improvement was symptomatic only; no radiological response of the mediastinal lesion to antibiotics was documented. He denied hemoptysis, weight loss, or night sweats, and had no history of malignancy, autoimmune disease, or tuberculosis exposure. On admission, vital signs were stable and physical examination was unremarkable apart from mildly reduced breath sounds over the right lower zone.

### Laboratory findings

Complete blood count (CBC), renal and hepatic panels, and coagulation profile were normal. A full tumor marker panel (carcinoembryonic antigen [CEA], squamous cell carcinoma antigen [SCC-Ag], neuron-specific enolase [NSE], cytokeratin 19 fragment antigen 21–1 [CYFRA 21-1], and alpha-fetoprotein [AFP]) was within normal limits. Inflammatory and infectious workup was notably unremarkable: procalcitonin (PCT) was <0.04 ng/mL, interleukin-6 (IL-6) was 2.67 pg/mL, and total IgE was 44.8 IU/mL, all within reference ranges. Sputum acid-fast staining, fungal culture, and bacterial culture were all negative. Serology for Epstein–Barr virus and cytomegalovirus was negative, and a respiratory virus panel (six pathogens) was negative. This profile argued against an active pyogenic, mycobacterial, fungal, viral, or allergic process.

### Imaging

**Figure 1:**
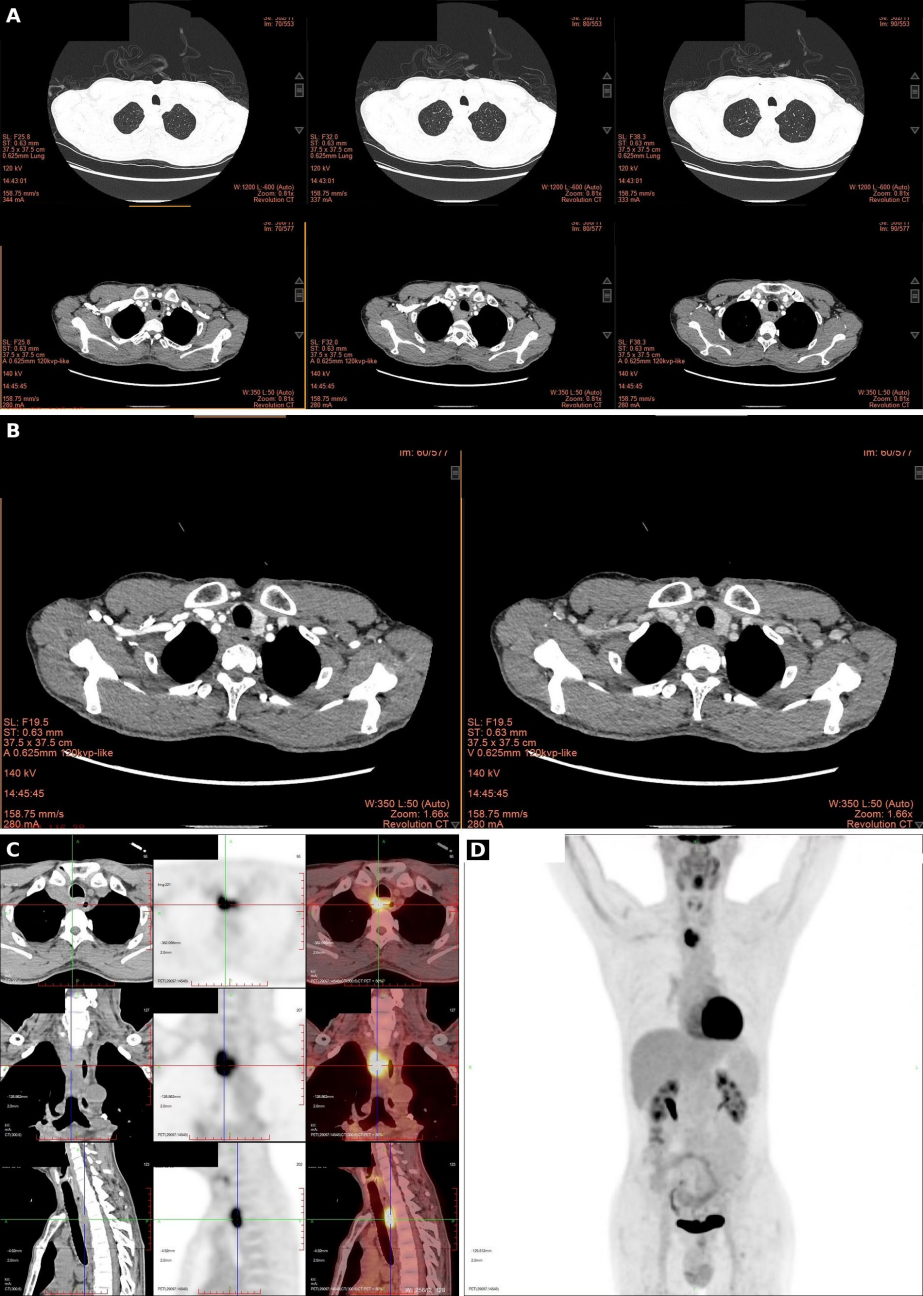
Pre-treatment diagnostic imaging. **(A)** Contrast-enhanced chest CT at contiguous levels (top: lung window; bottom: mediastinal window) showing an ill-defined soft-tissue lesion posterior to the trachea, within the tracheoesophageal groove of the superior mediastinum, with a poorly defined interface with the right lateral esophageal wall. **(B)** Magnified mediastinal-window images on the arterial phase (left) and venous phase (right), demonstrating heterogeneous enhancement of the mass and its close relationship to the adjacent great vessels. **(C)** Axial CT, PET, and fused [18F]FDG PET/CT images demonstrating intensely elevated FDG uptake (SUVmax 16.7) in the lesion, with no abnormal uptake in regional lymph nodes. **(D)** Whole-body maximum intensity projection (MIP) image showing a solitary mediastinal focus of intense uptake and no abnormal uptake elsewhere to suggest distant metastasis (physiological uptake is seen in the brain, myocardium, kidneys, bowel, and bladder).

Contrast-enhanced chest CT (non-ionic iodinated contrast medium, iodixanol, 3 mL/s, 80 mL; **Figures 1A, B**) demonstrated an ill-defined soft-tissue mass located posterior to the trachea, within the tracheoesophageal groove of the superior mediastinum, measuring 1.8×2.6×3.2 cm. The lesion showed mixed iso- to slightly hypodense attenuation (precontrast 17–41 HU) and heterogeneous enhancement on both arterial- and venous-phase images, with a poorly defined interface with the right lateral esophageal wall. The trachea and main bronchi showed no definite obstruction, the pericardium was not thickened, and no pathological lymphadenopathy was identified. Incidental findings included bronchiectasis with wall thickening and patchy opacity in the left lower lobe. Standard whole-body [18F]FDG PET/CT imaging was acquired approximately 60 min after intravenous administration of 250 MBq of [18F]FDG. The pre-injection blood glucose level was 6.0 mmol/L. SUVmax was normalized to total body weight (BW), and no delayed imaging was performed. The PET/CT images (**Figures 1C, D**) revealed strikingly intense [18F]FDG uptake with an SUVmax of 16.7, far exceeding mediastinal blood pool activity, with no abnormal uptake in regional lymph nodes or at distant sites. The PET/CT impression was a hypermetabolic mediastinal nodule, highly suspicious for malignancy.

### Bronchoscopy and endobronchial ultrasound

Bronchoscopy revealed a patent airway with no endobronchial lesion. EBUS-guided transbronchial needle aspiration (EBUS-TBNA) was attempted but did not yield a diagnostic specimen. A percutaneous core biopsy was therefore pursued to obtain larger-caliber tissue cores suitable for histological and immunohistochemical analysis.

### CT-guided biopsy

**Figure 2:**
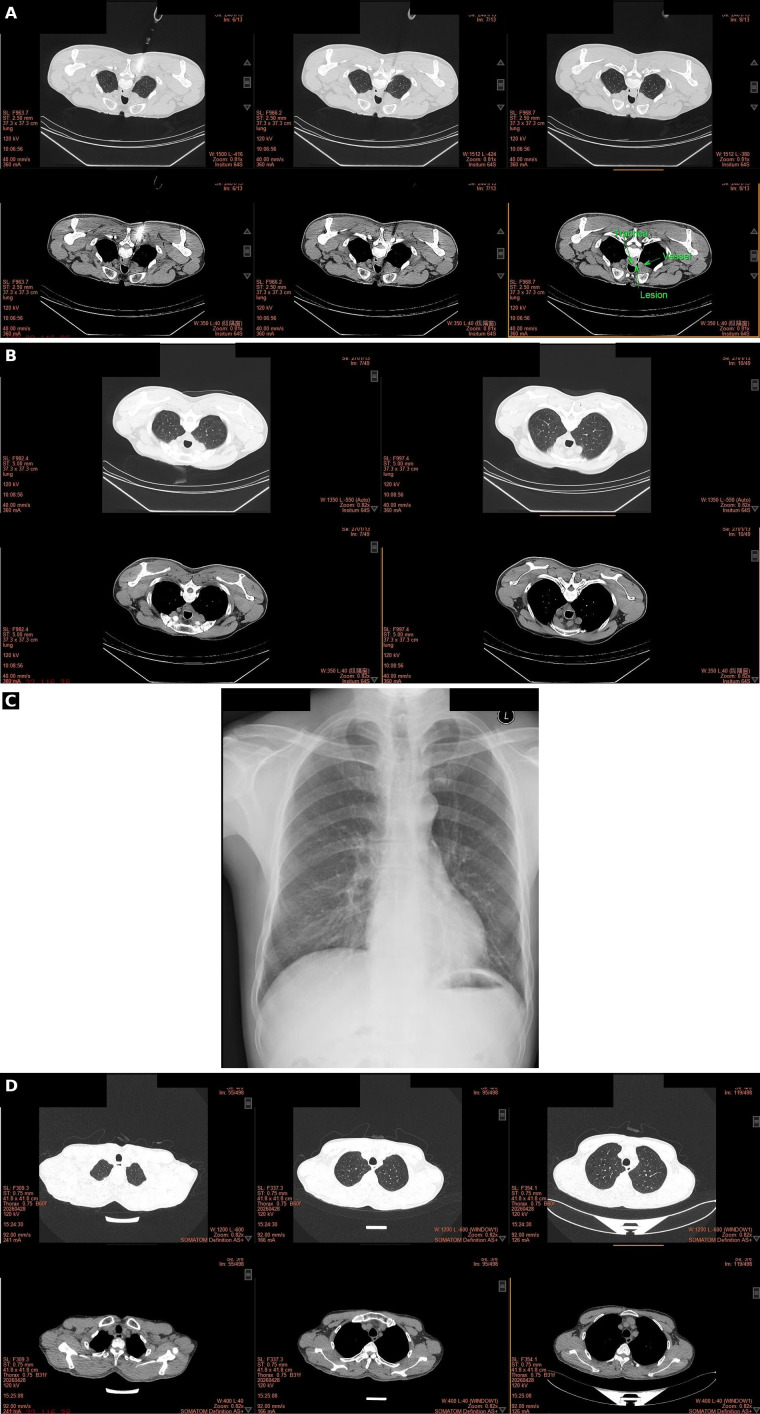
CT-guided biopsy, procedural safety, and follow-up. **(A)** CT-guided percutaneous core needle biopsy (top: lung window; bottom: mediastinal window) showing the coaxial needle traversing the chest wall toward the lesion; the final panel labels the spatial relationship of the target lesion to the adjacent trachea and vessel, illustrating the carefully planned trajectory used to sample the lesion safely. **(B)** Immediate post-procedural CT (paired lung and mediastinal windows) confirming the absence of pneumothorax, hemothorax, hemopericardium, or vascular injury. **(C)** Chest radiograph obtained on the day after biopsy, showing no pneumothorax and no pleural effusion, consistent with an uncomplicated procedure. **(D)** Follow-up chest CT approximately 2.5 months after biopsy (top: lung window; bottom: mediastinal window) demonstrating complete resolution of the mediastinal mass, with no residual soft-tissue abnormality in the previously affected region.

Following multidisciplinary review, CT-guided percutaneous core needle biopsy was performed. The lesion’s proximity to the trachea, superior vena cava, and aortic arch necessitated meticulous pre-procedural multiplanar trajectory planning. A paramedian anterior chest wall approach was selected using a 16-gauge coaxial system with real-time CT fluoroscopic guidance and patient breath-hold technique; the relationship of the needle tip to the trachea, adjacent vessel, and target lesion was monitored on paired lung- and mediastinal-window images at each stage of needle advancement (**Figure 2A**). Two diagnostic cores were obtained. Immediate post-procedural CT (**Figure 2B**) confirmed no pneumothorax, hemothorax, hemopericardium, or vascular injury, and a chest radiograph on the following day (**Figure 2C**) likewise showed no pneumothorax or pleural effusion. The patient remained hemodynamically stable throughout.

### Pathology

**Figure 3:**
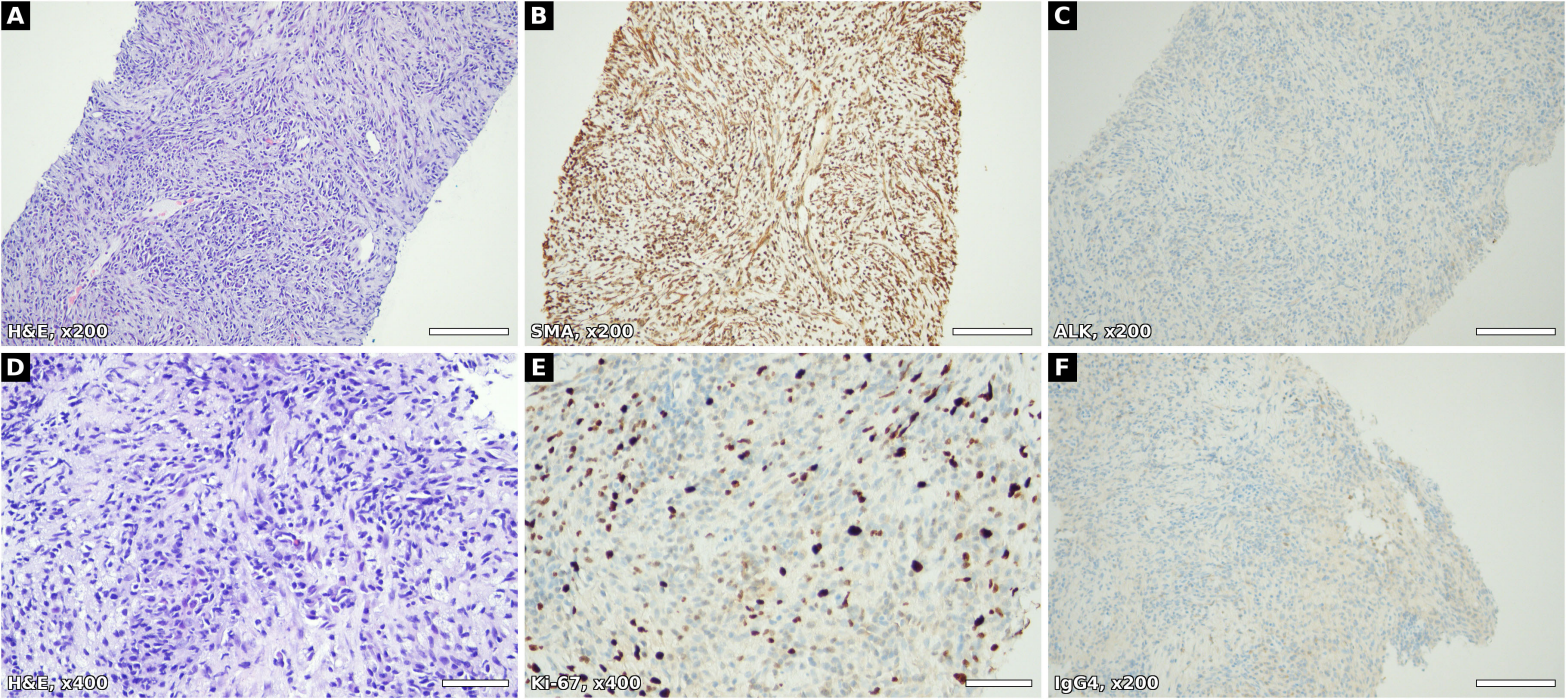
Histopathological and immunohistochemical findings. **(A)** Hematoxylin and eosin (H&E) stain (×200) showing fibrocollagenous tissue with bland spindle cells, a lymphoplasmacytic infiltrate, and foamy histiocytes, without cytological atypia or malignant cells. **(B)** Smooth muscle actin (SMA) (×200) demonstrating diffuse cytoplasmic positivity, indicating myofibroblastic differentiation. **(C)** ALK (×200) showing negative staining, excluding ALK-positive inflammatory myofibroblastic tumor but not excluding ALK-negative inflammatory myofibroblastic tumor. **(D)** H&E (×400) highlighting the bland, storiform-to-fascicular spindle-cell proliferation without atypical mitoses. **(E)** Ki-67 (×400) demonstrating a proliferative index of approximately 30%, labeling predominantly inflammatory cells and providing a histological correlate for the high FDG avidity. **(F)** IgG4 (×200) showing only sparse, focally weak positivity, well below the diagnostic threshold for IgG4-related disease.

Histopathology (**Figures 3A, D**) showed fibrocollagenous tissue with a dense lymphoplasmacytic infiltrate and focal foamy histiocyte aggregates, with no cytological atypia, necrosis, granulomas, or malignant cells. Immunohistochemistry (IHC; **Figures 3B, C, E, F**) demonstrated diffuse smooth muscle actin (SMA) positivity, indicating myofibroblastic differentiation, with negative ALK staining (excluding ALK-positive inflammatory myofibroblastic tumor) and negative desmin (arguing against a primary muscle-lineage neoplasm). CD68 highlighted the histiocytic infiltrate, corroborating the foamy histiocytes seen on hematoxylin and eosin staining. IgG4 staining was only focally and weakly positive, well below the diagnostic threshold for IgG4-related disease (fewer than 10 IgG4-positive cells per high-power field and an IgG4/IgG ratio below 40%). The Ki-67 proliferation index was approximately 30%; notably, the Ki-67-positive nuclei were distributed predominantly among the inflammatory cell population (lymphocytes and histiocytes), with only scattered positivity in the bland spindle (myofibroblastic) cells. The overall features favored an inflammatory myofibroblastic proliferation. However, because assessment was based on limited core-biopsy material, complete distinction from an ALK-negative inflammatory myofibroblastic tumor could not be made with absolute certainty.

### Clinical course and follow-up

Multidisciplinary tumor board (MDT) review concluded that the combined findings were most consistent with an inflammatory process. No antineoplastic, corticosteroid, immunosuppressive, or further targeted antimicrobial treatment was initiated, and active imaging surveillance was elected. Follow-up chest CT at approximately 2.5 months (**Figure 2D**) demonstrated complete disappearance of the mediastinal mass with no residual soft-tissue abnormality. In parallel with the radiological resolution, the patient’s dysphagia, cough, chest pain, and low-grade fever had all resolved completely, and he remained asymptomatic. A repeat chest CT in July 2026, approximately 2 months after the scan that first documented complete resolution, again showed no residual or recurrent mediastinal lesion. A clinical timeline is presented in **Table 1**.

**Table 1:** Clinical timeline of key events and decisions.

Timepoint	Event/investigation	Key finding/decision
Week −4	Onset of cough, right chest pain, low-grade fever, mild dysphagia; empirical antibiotics; partial symptomatic improvement	Outpatient treatment incomplete; referred to hospital
Admission	Vital signs stable; CBC, tumor markers, PCT, IL-6, IgE normal; sputum cultures and viral serology negative	No laboratory evidence of active infection; malignancy not established
Day 3	Contrast-enhanced chest CT	Ill-defined retrotracheal mediastinal mass (1.8×2.6×3.2 cm) in the tracheoesophageal groove
Day 8	[18F]FDG PET/CT	SUVmax 16.7; high suspicion for malignancy; tissue sampling recommended
Day 13	Bronchoscopy + EBUS-TBNA	No endobronchial lesion; aspiration non-diagnostic
Day 15	CT-guided core needle biopsy	Two cores obtained; no complications on post-biopsy CT or next-day chest radiograph
Day 16–18	Histopathology + MDT review	Inflammatory myofibroblastic proliferation; no malignant cells identified; surveillance elected
~Day 100	Supplementary IHC	ALK−, SMA+, IgG4 weakly+, Ki-67 ~30%; inflammatory process favored
~Week 10	Follow-up chest CT	Complete radiological resolution; patient asymptomatic
July 2026	Repeat chest CT (~2 months after documented resolution)	No residual or recurrent mediastinal lesion; sustained radiological resolution

## Discussion

This case highlights a clinically important diagnostic pitfall: an exceptionally FDG-avid mediastinal mass (SUVmax 16.7), with infiltrative margins and apparent esophageal contact, that followed a self-limiting course and completely resolved during imaging surveillance. The initial differential—esophageal carcinoma with mediastinal extension, lymphoma, and metastatic lymphadenopathy—was driven largely by the metabolic and morphologic findings, whereas the subsequent clinical course and serial imaging substantially lowered concern for malignancy.

The non-specificity of FDG uptake is central to understanding this case. FDG accumulates in metabolically active tissues, and proliferating inflammatory cells can generate intense signal (3, 7). Reported SUVmax values for inflammatory pseudotumors and inflammatory myofibroblastic lesions generally fall in the range of approximately 2 to 10 (4), and in a pathologically correlated series SUVmax ranged from 3.3 to 20.8, with stronger uptake in lesions of higher cellularity and proliferative index (8); the value of 16.7 in this case therefore lies at the upper extreme of what inflammatory myofibroblastic lesions may show and was a major driver of the initial suspicion of malignancy. Here, the Ki-67 index of approximately 30% provides a histological correlate for this intense uptake: a substantial fraction of cells within the lesion were actively cycling at the time of biopsy. Critically, the Ki-67-positive nuclei labeled predominantly the inflammatory cell population (lymphocytes and histiocytes) rather than the bland spindle cells. This distribution supports the interpretation that the elevated proliferative index was largely attributable to reactive inflammatory proliferation, although Ki-67 distribution alone cannot exclude a neoplastic process (7, 9).

### Interpretation as a self-limiting inflammatory myofibroblastic proliferation

The natural history was highly informative in interpreting this lesion. Inflammatory myofibroblastic tumor is classified as a neoplasm of intermediate biological potential, and ALK negativity does not by itself exclude this diagnosis because ALK-negative tumors are well recognized within the IMT spectrum (9–11). In the present case, complete disappearance of the lesion within approximately ten weeks in the absence of antineoplastic, corticosteroid, immunosuppressive, or further targeted antimicrobial therapy, followed by sustained absence of residual or recurrent disease on the July 2026 CT, supports a self-limiting inflammatory course. Rare spontaneous or treatment-associated regression has been described within the inflammatory myofibroblastic tumor spectrum (10, 12). Two histological observations also support this interpretation: the spindle (myofibroblastic) cells were cytologically bland and showed only scattered Ki-67 positivity, whereas most proliferative activity resided in the accompanying inflammatory cells. Together with the fibrocollagenous stroma, lymphoplasmacytic infiltration, and foamy histiocytes, the integrated clinicopathological findings favored an inflammatory myofibroblastic proliferation with an organizing, self-limiting course. Nevertheless, because only core-biopsy material was available and histological overlap with ALK-negative inflammatory myofibroblastic tumor may occur, we cannot establish this distinction with absolute certainty. The diagnosis is therefore presented as the interpretation best supported by the combined pathology and serial clinical course rather than as a categorical exclusion of neoplasia.

### Differential diagnosis

The absence of malignant cells in two core-biopsy specimens, together with subsequent complete radiological resolution and sustained absence of recurrence on repeat CT, substantially reduced the likelihood of malignancy; however, the limitations of core-biopsy sampling preclude absolute exclusion of an unsampled malignant component. Active infection was systematically addressed: procalcitonin was very low (<0.04 ng/mL) and interleukin-6 was normal; sputum acid-fast staining and fungal culture were negative; and Epstein–Barr virus, cytomegalovirus, and a respiratory virus panel were all negative. A normal total IgE also made an allergic process less likely. The histological findings did not meet established criteria for IgG4-related disease (13). The partial improvement after empirical antibiotics was limited to symptoms, and no interval imaging documented radiological regression during antimicrobial treatment. The subsequent complete disappearance of the lesion occurred during surveillance without further targeted antimicrobial, corticosteroid, immunosuppressive, or antineoplastic therapy. Accordingly, although a contribution from the preceding antibiotic treatment cannot be entirely excluded, the overall temporal course favors a self-limiting inflammatory process.

### Did the biopsy itself cause the regression?

A legitimate concern is whether the core biopsy, rather than the natural course of the lesion, precipitated its disappearance. We consider a major biopsy-induced mechanical effect unlikely for several reasons. Only two thin cores were obtained from a solid lesion measuring approximately 3 cm, and the lesion was solid rather than cystic or abscess-like, with no evacuable fluid or necrotic component on imaging or histology. Rare spontaneous or treatment-associated regression has been reported within the inflammatory myofibroblastic tumor spectrum (10, 12), making a self-limiting course biologically plausible. Nonetheless, because this is a single case, a contribution from biopsy-related local injury or an incompletely understood immunological effect cannot be excluded, and this possibility is acknowledged explicitly.

### Procedural considerations

The safe completion of CT-guided core biopsy despite proximity to the trachea, superior vena cava, and aortic arch illustrates that vascular adjacency need not preclude percutaneous sampling. Meticulous multiplanar trajectory planning, a coaxial technique limiting pleural passages, real-time fluoroscopic guidance with breath-hold, continuous visualization of the needle tip relative to adjacent vessels, and immediate post-procedural imaging are key to mitigating risk (5, 6). Large contemporary series have confirmed that image-guided percutaneous core-needle biopsy of mediastinal masses is safe and achieves a high diagnostic yield, with major complications in well under 1% of cases (14). In this patient, obtaining tissue that showed no malignant cells and integrating the biopsy findings with serial imaging allowed immediate surgery to be avoided.

### Management implications

The decision to defer surgery in favor of surveillance was made after malignancy and active infection had become less likely on the basis of tissue sampling, laboratory testing, imaging, and multidisciplinary review. The subsequent complete radiological resolution, together with sustained absence of recurrence on the July 2026 CT, supported that management decision. This case suggests that active imaging follow-up may be appropriate in carefully selected patients with a non-malignant core-biopsy result when close clinical and radiological monitoring is feasible and any progression would prompt immediate re-evaluation.

### Limitations

The biopsy cores were small, raising a theoretical concern about sampling error; however, complete resolution without antineoplastic therapy and sustained absence of recurrence on repeat CT provide additional evidence against an unsampled malignant component. Microbiological culture and special stains were not performed on the biopsy tissue itself, although the comprehensive sputum and serological workup and the normal PCT/IL-6 indirectly argue against active infection. Serum IgG4 was not measured, but tissue IgG4 staining was clearly below the diagnostic threshold. Although a repeat chest CT in July 2026 confirmed sustained resolution approximately 2 months after the first scan documenting disappearance of the mass, longer-term follow-up remains limited. Continued annual imaging surveillance is recommended.

## Conclusion

An inflammatory myofibroblastic proliferation in the mediastinum may exhibit extreme FDG avidity (SUVmax 16.7) that is radiologically indistinguishable from malignancy. An elevated reactive Ki-67 index may provide a histological correlate for the high metabolic signal, whereas complete spontaneous resolution sustained on repeat CT supports a self-limiting inflammatory course. Because core-biopsy material is limited and ALK-negative inflammatory myofibroblastic tumor cannot be excluded with absolute certainty, clinicopathological interpretation should remain cautious. A structured workup that makes malignancy and active infection less likely, combined with short-interval imaging surveillance, may allow carefully selected patients to avoid unnecessary surgery or empirical oncological treatment. Intense FDG uptake alone should not be equated with malignancy.

## Patient perspective

When first told that imaging suggested a possible cancer, the patient experienced considerable anxiety and had anticipated needing major surgery. He was reassured by the biopsy result and chose to follow the recommendation for close imaging surveillance rather than immediate operation. He described considerable relief when the follow-up scan showed that the lesion had disappeared. He felt that avoiding an unnecessary operation had been the right decision for him.

## Data Availability

The original contributions presented in the study are included in the article/**Supplementary Material**. Further inquiries can be directed to the corresponding author.
