# Supplementary material for: Case Report: a hypermetabolic mediastinal mass (SUVmax 16.7) mimicking malignancy with spontaneous resolution: an inflammatory myofibroblastic proliferation and diagnostic pitfall
Source: Front Oncol. 2026 Aug 5;16:1901288. doi: 10.3389/fonc.2026.1901288 (PMC13485795; doi:10.3389/fonc.2026.1901288)
Supplement: Supplementary file 1 [file DataSheet1.pdf]

CARE Checklist (2013) of information to include when writing a case report

Completed for the manuscript: *“Case Report: A Hypermetabolic Mediastinal Mass (SUVmax 16.7) Mimicking Malignancy With Spontaneous Resolution: An Inflammatory Myofibroblastic Proliferation and Diagnostic Pitfall”*

Note: As the manuscript uses continuous pagination without fixed line numbers, locations are given by section (and figure/table) rather than line number, in line with CARE guidance.

Topic	Item	Checklist item description	Reported in Section
**Title**	**1**	The diagnosis or intervention of primary focus followed by the words “case report”	Title
**Key Words**	**2**	2 to 5 key words that identify diagnoses or interventions in this case report, including “case report”	Keywords
**Abstract**	**3a**	Introduction: What is unique about this case and what does it add to the scientific literature?	Abstract – Background
	**3b**	Main symptoms and/or important clinical findings	Abstract – Case Summary
	**3c**	The main diagnoses, therapeutic interventions, and outcomes	Abstract – Case Summary
	**3d**	Conclusion—What is the main “take-away” lesson(s) from this case?	Abstract – Conclusion
**Introduction**	**4**	One or two paragraphs summarizing why this case is unique (may include references)	Introduction
**Patient Information**	**5a**	De-identified patient specific information	Case Description – Clinical presentation
	**5b**	Primary concerns and symptoms of the patient	Case Description – Clinical presentation
	**5c**	Medical, family, and psycho-social history including relevant genetic information	Case Description – Clinical presentation
	**5d**	Relevant past interventions with outcomes	Case Description – Clinical presentation (prior empirical antibiotics)
**Clinical Findings**	**6**	Describe significant physical examination (PE) and important clinical findings	Case Description – Clinical presentation & Laboratory findings
**Timeline**	**7**	Historical and current information from this episode of care organized as a timeline	Table 1
**Diagnostic Assessment**	**8a**	Diagnostic testing (such as PE, laboratory testing, imaging, surveys)	Case Description – Laboratory findings; Imaging; Bronchoscopy and EBUS; Pathology (Figures 1, 3)
	**8b**	Diagnostic challenges (such as access to testing, financial, or cultural)	Case Description – Bronchoscopy and EBUS (non-diagnostic aspiration); CT-guided biopsy (vascular proximity)
	**8c**	Diagnosis (including other diagnoses considered)	Case Description – Pathology; Discussion – Differential diagnosis
	**8d**	Prognosis (such as staging in oncology) where applicable	Discussion – self-limiting course; Conclusion
**Therapeutic Intervention**	**9a**	Types of therapeutic intervention (such as pharmacologic, surgical, preventive, self-care)	Case Description – Clinical course and follow-up (no antineoplastic, corticosteroid,

Topic	Item	Checklist item description	Reported in Section
			immunosuppressive, or further targeted antimicrobial therapy; surveillance)
	**9b**	Administration of therapeutic intervention (such as dosage, strength, duration)	Case Description – Clinical presentation (prior empirical antibiotics); Clinical course and follow-up (no further targeted antimicrobial, antineoplastic, corticosteroid, or immunosuppressive therapy)
	**9c**	Changes in therapeutic intervention (with rationale)	Case Description – Clinical course and follow-up (not applicable)
**Follow-up and Outcomes**	**10a**	Clinician and patient-assessed outcomes (if available)	Case Description – Clinical course and follow-up; Patient Perspective
	**10b**	Important follow-up diagnostic and other test results	Case Description – Clinical course and follow-up (Figure 2D; July 2026 repeat CT showing sustained resolution); Table 1
	**10c**	Intervention adherence and tolerability (How was this assessed?)	Case Description – Clinical course and follow-up (surveillance adhered to; asymptomatic)
	**10d**	Adverse and unanticipated events	Case Description – CT-guided biopsy (no complications); Clinical course (spontaneous resolution)
**Discussion**	**11a**	A scientific discussion of the strengths AND limitations associated with this case report	Discussion; Discussion – Limitations
	**11b**	Discussion of the relevant medical literature with references	Discussion (refs 1–14)
	**11c**	The scientific rationale for any conclusions (including assessment of possible causes)	Discussion – Interpretation as a self-limiting inflammatory myofibroblastic proliferation; Differential diagnosis; Did the biopsy itself cause the regression?
	**11d**	The primary "take-away" lessons of this case report (without references) in a one paragraph conclusion	Conclusion
**Patient Perspective**	**12**	The patient should share their perspective in one to two paragraphs on the treatment(s) they received	Patient Perspective
**Informed Consent**	**13**	Did the patient give informed consent? Please provide if requested	Ethics Statement – waiver of informed consent granted (approval no. 2026(106)); available on request

This manuscript was prepared following the CARE guidelines (https://www.care-statement.org).
Author completing this checklist: Zhe Li, on behalf of all authors. Date: July 8, 2026.
